# Functional Parathyroid Cyst: A Rare Cause of Malignant Hypercalcemia with Primary Hyperparathyroidism—A Case Report and Review of the Literature

**DOI:** 10.1155/2012/851941

**Published:** 2012-02-15

**Authors:** Areej Khan, Yusra Khan, Shahzad Raza, Ghulam Akbar, Monis Khan, Nauman Diwan, Wajih Rizvi

**Affiliations:** ^1^Department of Internal Medicine, Brookdale University Hospital and Medical Center, Brooklyn, NY 11212, USA; ^2^Bronx Lebanon Hospital, New York, NY 10457, USA; ^3^McMaster University, Hamilton, ON, Canada L8N 3Z5; ^4^Raritan Bay Medical Center, New Jersey, NJ 08857, USA; ^5^Department of R-Endocrinology, Division of Endocrinology and Metabolism, Robertwood Johnson University of Medicine and Dentistry (UMDNJ), Hamilton, NJ 08690, USA

## Abstract

Parathyroid cysts are rare lesions found in the neck and anterior mediastinum. They are often nonfunctional (>90%) and rarely in the functional form. This paper discusses a case of severe hypercalcemia (23 mg/dL) secondary to a rare functional parathyroid cyst. The patient was later found to have a hemorrhagic cyst with compression of the right recurrent laryngeal nerve. Preoperative diagnosis of the lesion was parathyroid carcinoma. However, reexploration of the parathyroid mass along with microscopic study confirmed the diagnosis of a parathyroid cyst. Following cystectomy, the patient restored her baseline functional status with preservation of the right recurrent laryngeal nerve. Postoperative followup three years later showed no evidence of cyst recurrence. This paper illustrates the rare presentation of parathyroid functional cysts with severe hypercalcemia and primary hyperparathyroidism. Physicians should be aware of the presence of hemorrhage, inflammation, and compressive symptoms in these cysts which mimic parathyroid carcinoma. These patients should be managed with aggressive medical and surgical intervention.

## 1. Introduction

Parathyroid cysts, first reported in 1905, are rare lesions found in the neck and anterior mediastinum [1–4]. They often manifest as asymptomatic neck masses [4] that are discovered as incidental findings during neck surgeries or imaging procedures. These cysts can cause mechanical compression of surrounding structures that lead to wheezing from airway compromise, dysphagia, and voice hoarseness from recurrent laryngeal nerve palsy. Innominate vein thrombosis [5] is also another clinical finding when multiple groups of parathyroid cysts are involved. Currently, there are less than 300 documented cases of parathyroid cysts, making up only 0.6% of all reported thyroid and parathyroid lesions [3].

Crile Jr. and Perryman [6] reported the first case on parathyroid cyst, in 1953, that was diagnosed after fine needle aspiration of the parathyroid cyst. Since then, the literature has focused on two broad categories of parathyroid cysts: functional and nonfunctional. Whereas functional cysts comprise 10% of parathyroid cysts [7] and occur more commonly in males, nonfunctional cysts encompass the other 90% and occur more commonly in females [7, 8]. In contrast to normal parathyroid function seen in cases of nonfunctional cysts, functional cysts are clinically associated with primary hyperparathyroidism and elevated calcium levels exceeding 13 mg/dL. While nonfunctional cysts frequently occur in the inferior parathyroid glands, the location of functional cysts is variable. Symptoms depend on the location of functional cysts. As with nonfunctional parathyroid cysts, patients with functional cysts may be asymptomatic or present with dyspnea, dysphagia, or dysphonia depending on the structures compressed within the anterior mediastinum. Cystic hemorrhage during surgical intervention is an additional known complication. Due to their hemorrhagic presentation, functional cysts can be misdiagnosed as malignant lesions. Functional cysts are diagnosed by an ultrasound and through measurements of PTH level in the aspirated cystic fluid. Overall, management involves aggressive fluid hydration to treat hypercalcemic crisis as well as surgical intervention. In this paper, we present a case of a patient with a functional parathyroid cyst whose clinical signs and symptoms resemble parathyroid carcinoma. 

## 2. Case Presentation

A 58-year-old Asian female with a five-year history of depression presented to the emergency room with progressive generalized weakness, nausea, vomiting, and weight loss over the past three months. She stated her symptoms recently started worsening over the past two weeks. She complained of increased thirst, generalized bone pain, and weight loss of greater than 20 pounds in the past one month. Despite being on antidepressants, the patient presented with symptoms of depression. She denied any history of hypercalcemia, hyperparathyroidism, or cancer. She denied any history of smoking, alcohol, or use of illicit drugs. Family history was unremarkable.

On physical examination, she was afebrile, blood pressure 110/73, and pulse 82 beats per minute. Tongue was dry on examination. Neck revealed a 4 × 4 cm fluctuant smooth contour nodule that was nontender on palpation and moved with deglutition. The patient was subsequently worked up and found to have hypercalcemia, (serum calcium level, 23.3 mg/dL) (normal range 8.6–10.6 mg/dL), (phosphorous, 3.1 mg/dL) (normal range 2.4–4.4 mg/dL), albumin 2.8 g/dL (normal range 3.5–5.0 g/dL), BUN 52 mg/dL (normal range 7–21 mg/dL), creatinine 3.8 mg/dL (normal range 0.7–1.5), blood glucose 91 mg/dL (normal range 65–105 mg/dL) Blood work for ANA, Rheumatoid factor, ACTH, cortisol, liver functions were unremarkable.

**Figure 1:**
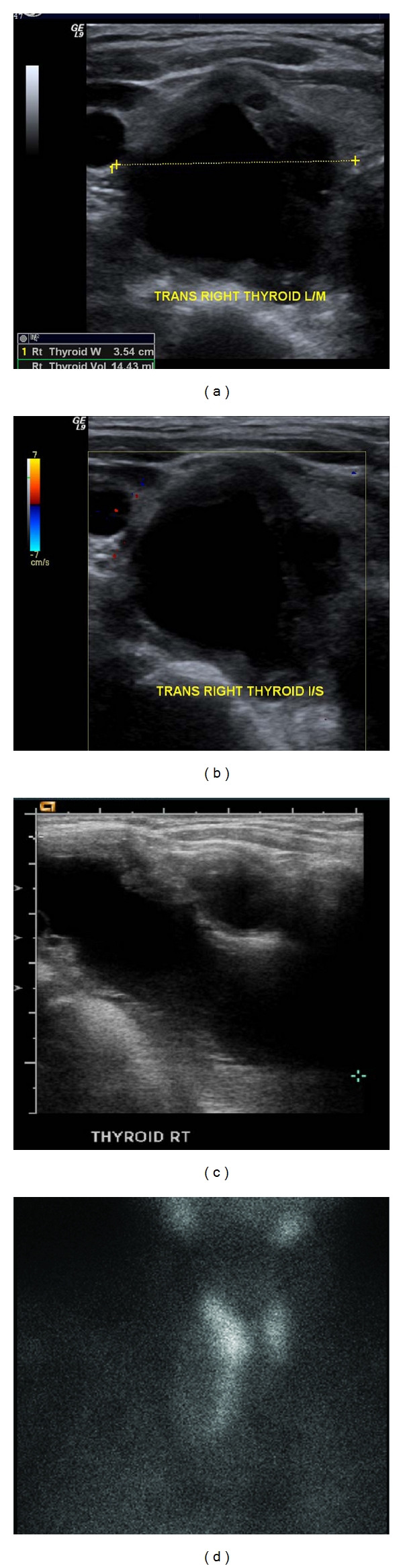
(a, b, c) Thyroid ultrasound showing large complex cystic mass within or adjacent to the right lobe of the thyroid gland measuring up to 4.5 cm representing thyroid versus parathyroid lesion. (d) Parathyroid scan shows increased uptake in the lobes of thyroid gland. “MIBI avid lesion with retained activity in the periphery.”

Intravenous hydration and loop diuretics were administered to promote kalciuresis. After the patient was resuscitated, further evaluation revealed plasma level of intact parathyroid hormone (PTH) to be 1364 pg/mL (normal 10–65 pg/mL). Thyroid ultrasonography showed 3.5 × 4.5 × 3.2 cm complex cystic and solid lesion in the right thyroid gland. A parathyroid scan with Technitium-99-sestamibi scintigraphy revealed an “avid lesion with retained activity in the periphery” (Figure 1). Fine needle aspiration of the cold mass revealed hemorrhagic fluid without evidence of malignant cells in the tissue. The tissue stained for PTH was negative for thyroid transcription factor-1 (TTF1). A CT scan of head and abdomen was unremarkable. Preoperatively, dual energy X-ray absorptiometry bone mineral density study (DEXA) showed osteopenia of the lumbar spine, left hip, and left femoral neck.

The patient underwent surgical exploration for the cyst twice by two different surgeons on two separate occasions. The first surgery was stopped half way with the postoperative diagnosis of parathyroid carcinoma that was not operable due to 2-3 cm mass underneath the strap muscles that was adherent to the right side of the trachea and dense fibrotic tissue (initially suspected to be a tumor) along the right side of the neck. The patient's condition was further complicated by paralysis of the right vocal cord from the first surgical exploration.

In the second surgical exploration, the following steps were taken.

The previous incision was reopened and the flap was raised in the superior direction with use of cautery. Sternohyoid muscles were ligated. There was dense inflammatory tissue involving the sternothyroid muscle, carotid, and jugular veins. The sternothyroid muscle was firmly adherent to the thyroid. Dissection began with incision of the median raphe and the isthmus muscle and proceeded in the medial to lateral direction. The thyroid was then elevated at the level of trachea medially and the right recurrent laryngeal nerve was identified inferiorly. 

**Figure 2:**
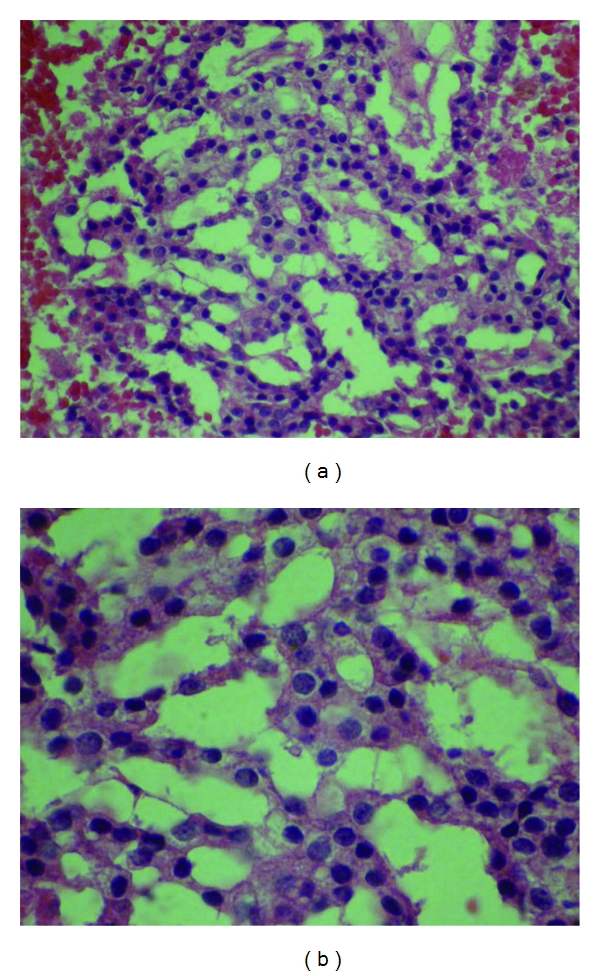
(a, b)  Histopathological examination of the cyst revealed a cyst wall composed of fibroadipose tissue containing islands of parathyroid tissue ((a) HE stain 100x, (b) HE stain 400x).

The patient was preoperatively documented to have paralysis of the right vocal cord. However, the right recurrent laryngeal nerve was later found to be intact after stimulation with a threshold of 1.7 mA. Following stimulation, the intact recurrent laryngeal nerve was dissected into the superior mediastinum and superiorly in its entry at the cricothyroid membrane. There was dense inflammatory tissue adhering to both the strap muscles and the right lobe of thyroid. Suspecting a tumor, an incision was made along the borders of the mass. In the attempt to resect the tumorlike mass, the right lobe of thyroid adhering to the dense mass was resected as well. The frozen section of the dense mass revealed only collagen and fibrous tissue. The sternothyroid muscle, the carotid artery, and the jugular vein were then freed from the dense inflammatory tissue in an en bloc dissection. Further into the dissection, a cystic mass with a glistening capsule was then found in the superior mediastinum near adjacent parathyroid tissue. The cyst was removed from the superior mediastinum and the specimen was sent for frozen section. Microcopic analysis of the cystic mass confirmed the diagnosis of an enlarged hypercellular parathyroid (330 mg). In addition to the hypercellular parathyroid tissue, the specimen contained hyalinized fibrous connective tissue scar, right thyroid lobe, and the cystic wall. Within the fibrous tissue there was hemorrhage and residual hypercellular parathyroid tissue with osteoid focus (Figure 2).

After surgery, the patient had variable parathyroid hormone levels between 110 and 292 pg/mL (normal range 10–65 pg/mL) along with hypocalcaemia. The patient underwent MIB1 scintigraphy for detection of aberrant parathyroid tissue causing elevated parathyroid hormone levels. Postoperative MIBI scintigraphy study was negative for parathyroid tissue.

The patient was prescribed calcium 1 gram per day. Her symptoms of polyuria, polydipsia, and depression subsequently subsided after surgery. The patient restored her original voice with resolution of voice hoarseness. Renal functions improved (creatinine 1.1 mg/dL) (normal range 0.7–1.5 mg/dL). Repeat neck ultrasonography and 99 mTc methoxyisobutylisonitrile (MIBI) scintigraphy after six months did not reveal remaining active parathyroid tissue. However, she developed low T4 (0.5 ng/dL) and elevated TSH (14.2 uIU/mL) because of hemithyroidectomy. She was then maintained on Synthroid 50 mcg orally every day. After a 3-year follow-up, the patient remained asymptomatic.

## 3. Discussion

Functional parathyroid cysts are an extremely rare cause of primary hyperparathyroidism. The criteria defining a functional parathyroid cyst include the following: (1) preoperative clinical and biochemical evidence of hyperparathyroidism, (2) intraoperative identification of normal remaining parathyroid glands, (3) histologic identification of parathyroid tissue within the cyst wall, and (4) postoperative correction of hypercalcemia. All of these features were present in our case. Moreover, we noticed severe parathyrotoxic crises with hypercalcemia (>23 mg/dL) (normal range 8.6–10.6 mg/dL), along with acute symptoms. In our case, the symptoms were reversed with the correction of hypercalcemia. To date, this is the highest serum calcium reported in a functioning parathyroid cyst with primary hyperparathyroidism [8].

This case illustrates that functional parathyroid cysts are rarely diagnosed; however timely management can improve the outcome of these patients. As presented in our case, the patient had a preoperative diagnosis of a functioning parathyroid cyst that mimicked a preoperative parathyroid carcinoma. The strong clinical similarities between parathyroid cyst and parathyroid carcinoma increase the risk of misdiagnosis. The rarity in the number of documented cases of parathyroid cysts may be from a lack of recognition of these cysts in clinical practice. Due to their rare presentation and clinical similarities to parathyroid carcinoma, parathyroid cysts require greater scrutiny and a better understanding of the disease by medical practitioners for an accurate and timely diagnosis.

It is unclear how parathyroid cysts develop and what makes them functional versus nonfunctional. However, five major theories suggest that parathyroid cysts are developed from embryological remnants of the third or fourth branchial clefts, coalescence of preexisting microcysts, simple retention of parathyroid secretions, vestigial remnants of the Kürsteiner canals [8–10] that are present during the fetal life in the parathyroid glands or cystic degeneration in preexisting adenomas [9, 10]. No one theory completely explains their origin, and many factors probably contribute to their etiology. The foci of hemorrhage noticed in our case suggest the possibility of parathyroid cyst from degenerating adenoma. 

Interestingly, hemorrhage in a parathyroid cyst has been associated with hypercalcemic crisis in previous reports [9] and our case further strengthens the evidence of direct association of hemorrhage and hypercalcemia [10].

The management of our case was very challenging, requiring a multidisciplinary approach. Initially, the patient had severe parathyrotoxic crises that required aggressive medical treatment. In addition to volume expansion, loop diuretics such as furosemide, control volume overload, and promotion of calcium diuresis proved to be very effective with intravenous pamidronate, a bisphosphonate. our patient responded to the medical management and did not require calcium-free hemodialysis for the management of hypercalcemia.

Functional parathyroid cysts are not only secretary but sometimes also cause local compression producing symptoms such as recurrent nerve paresis and hemorrhage [11]. These two features were particularly dominating in our case, thus indicating that surgical removal of parathyroid cyst is required in this patient. Manouras et al. [10] have suggested that during surgical exploration, surgeons should identify all parathyroid glands to rule out multiglandular disease, including cysts coexisting with adenomas, cysts existing in different parathyroid glands, adenomas coexisting with hyperplasia of the other parathyroid glands, double parathyroid adenomas which would result in recurrent hyperparathyroidism after surgery.

## 4. Conclusion

Although the disease is rarely encountered in clinical practice, physicians should be aware of the importance of early disease recognition and prompt management of patients presenting with severe hypercalcemia. In this paper, we noticed that management of a patient with primary hyperparathyroidism from a functional parathyroid cyst was successful due to early medical and surgical intervention. Aggressive medical treatment should always be instituted immediately with simultaneous diagnostic workup. Surgical intervention should then be performed as soon as hypercalcemia is controlled. This case demonstrated that patient can have compressive symptoms in addition to elevated serum calcium; therefore, early operation is mandatory before serious complications occur from involvement of the cardiovascular, central nervous, renal, or neuromuscular system.
